# 사물인터넷 기반 모바일 건강관리 애플리케이션의 한국 노인 대상 사용자 참여도에 따른 효과: 준실험 연구의 2차 분석

**DOI:** 10.7586/jkbns.24.034

**Published:** 2025-02-21

**Authors:** Jeongeun Choi, Hyeonmi Cho, Jo Woon Seok, Hyangkyu Lee

**Affiliations:** 1 Mo-Im Kim Nursing Research Institute, College of Nursing, Yonsei University, Seoul, Korea; 1 연세대학교 간호대학 김모임간호학연구소; 2 College of Nursing and Brain Korea 21 FOUR Project, Yonsei University, Seoul, Korea; 2 연세대학교 간호대학 4단계 두뇌한국(BK)21 S-L.E.A.P 미래간호인재 교육연구단; 3 Research Institute of AI and Nursing Science, College of Nursing, Gachon University, Incheon, Korea; 3 가천대학교 간호대학 AI간호과학 연구소; 4 College of Nursing, Ewha Womans University, Seoul, Korea; 4 이화여자대학교 간호대학

**Keywords:** Aged, Artificial intelligence, Mobile applications, Delivery of health care, 노인, 인공지능, 모바일 애플리케이션, 건강 서비스 제공

## Abstract

**Purpose:**

This study aimed to evaluate the effect of the TouchCare system, a digital health management system utilizing the internet of things (IoT), based on the usage levels of older adults.

**Methods:**

This is a secondary analysis of data from a quasi-experimental study examining the effects of an IoT-based digital healthcare system. Participants were equipped with the TouchCare application, a touch-tag, and context-aware artificial intelligence. Data on cognitive function, frailty, depressive symptoms, nutritional status, and fall efficacy were collected at baseline and after six months of using the system. The participants were divided into a high-engagement group (n = 22) and a low-engagement group (n = 24) based on how many days they used the application during the study. We used descriptive statistics, the paired t-test, the independent-samples t-test, and two-way mixed analysis of variance.

**Results:**

In total, 46 participants completed the evaluations (mean age, 76.6 years). Two-way mixed analysis of variance revealed no significant group-by-time interaction for cognitive function (*p* = .184), frailty (*p* = .338), depressive symptoms (*p* = .543), and nutritional status (*p* = .589). There was no significant difference in fall efficacy between the two groups (*p* = .091). The high-engagement group exhibited significant improvements in visuospatial and executive functions on the Montreal Cognitive Assessment (*p* = .029).

**Conclusion:**

The IoT-based mobile health management application demonstrated benefits in improving cognitive health among older adults. The findings suggest that active engagement with healthcare technology can positively affect health in this population, emphasizing the need for continuous support from nurses as health providers.

## 서론

### 1. 연구의 필요성

65세 이상 인구수는 전 세계적으로 2021년 7억 6,100만 명에서 2050년에는 16억 명으로 두 배 이상 증가할 것으로 예상된다[1]. 한국은 고령화 속도가 매우 빨라 2050년에 세계에서 가장 고령화된 국가가 될 것으로 전망되며[1], 이는 노인 인구의 건강관리를 효과적으로 지원하기 위한 시스템 구축의 중요성을 시사한다.

인구 고령화와 함께 증가하고 있는 노인의 건강 문제는 점차 복합적이고 다면적인 양상을 보이며[2], 대표적으로 노쇠, 인지기능 저하, 우울, 영양 불균형, 낙상이 해당된다. 노쇠는 노화로 인하여 외부 스트레스나 환경 변화에 대처하고 회복할 수 있는 능력이 저하되는 노인성 복합 증후군이다[3]. 전 세계 노인의 약 16%가 노쇠를 경험하는 것으로 보고되어, 최근 건강관리의 주요 과제로 부각되고 있다[4]. 인지기능 저하는 치매로의 진행 가능성을 예측하는 주요 지표로, 노인의 독립적인 생활과 삶의 질을 위해 조기 관리가 필수적이다[5]. 우울은 노년기에 가장 흔히 나타나는 정신 건강 문제로, 신체활동 감소와 사회적 고립을 초래하며 삶의 질을 크게 저하시킬 수 있다[6]. 노화로 인한 식욕 감소와 소화 기능 저하는 영양 불균형을 초래할 수 있으며, 이는 근감소증, 노쇠, 사망률 증가의 위험 요인이다[7]. 낙상은 신체적 손상뿐 아니라 심리적 두려움을 유발하며, 낙상에 대한 두려움이 높아질 경우 신체 활동 감소, 기능 저하로 이어져 노인의 독립성을 위협할 수 있다[8]. 이처럼 복합적인 노인의 건강 문제를 예방하고 관리하기 위해서는 통합적 접근을 기반으로 일상에서 건강 행동을 촉진할 수 있는 시스템이 필요하다.

최근 사물인터넷(internet of things, IoT)은 일상에서 건강 행동을 촉진할 수 있는 효과적인 시스템으로 부상하고 있다. 사물인터넷은 정보, 데이터, 자원을 자동으로 구성하여 상황과 환경의 변화에 대응하는 개방적이고 포괄적인 지능형 네트워크로 정의된다[9]. 헬스케어 분야에서 IoT의 이점은 높은 응용 가치를 보이며, 전 세계 헬스케어 시장에서 IoT의 가치는 2020년부터 2027년까지 연평균 13.2%의 성장률을 기록할 것으로 예상된다[10]. IoT는 일상적으로 사용하는 물건들이 네트워크로 연결되어 활동 지표를 생성하고, 이를 바탕으로 행동 패턴을 분석하여 건강 행동을 증진하는 데 활용되고 있다[11]. 예를 들어, 주거 공간에 설치된 센서와 스마트폰으로 수집된 생체 정보는 실시간 건강 상태 모니터링, 예방적 건강관리, 특정 응급 상황의 판단과 대응 서비스에 이용되고 있다[12].

특히 스마트폰과 IoT를 접목한 건강관리 서비스는 한국의 높은 모바일 기기 보급률과 함께 시공간의 제약이 적고 휴대성과 편리성이 높다는 장점이 있다[13]. IoT 기반 건강관리 서비스는 수집된 데이터를 근거로 건강 행동을 유도하여 자가 관리 역량을 강화할 수 있다[14]. 또한, 치료의 접근성 증진, 의료 비용 감소, 질병의 조기 발견, 신속한 의료 서비스 제공으로 노인의 삶의 질을 증진할 수 있다[15]. 이처럼 IoT 기술을 적극적으로 활용한 건강관리 중재는 노인에게 건강관리의 동기를 제공하고 효율성을 높여, 의료 재정 부담을 완화하고 독립적인 노후를 지원하는 데 기여할 수 있다.

터치케어 시스템(TouchCare, DNX Co. Ltd, Gyeonggi-do, Korea)은 터치케어 모바일 애플리케이션, 터치 태그 센서, 상황인지 대화형 인공지능으로 구성된 노인을 대상으로 한 IoT 기반 디지털 건강관리 시스템이다[16]. 지역사회 노인 14명을 대상으로 시행된 예비 연구에서 5개월 동안 본 시스템 중재를 적용하여 사용 전과 후의 건강 평가를 수행한 결과 영양상태와 낙상 효능감이 유의하게 개선되었다[16].

사용자의 참여도는 IoT 기술을 활용한 헬스케어 서비스 중재의 효과를 결정짓는 핵심적 요인으로, 디지털 건강관리 시스템의 이점은 사용자가 적극적으로 참여할 때 극대화될 수 있다[17,18]. 사용자 참여도와 건강 결과의 관계를 분석한 33개의 논문을 체계적 문헌 고찰한 연구에서는 사용자 참여도가 건강 관련 지표들(체중 관리, 흡연 여부, 신체활동, 과일 및 채소의 섭취)의 개선과 유의미한 관련이 있는 것으로 나타났다[18]. 국내에서도 IoT를 접목한 디지털 건강관리 서비스가 점차 증가하는 가운데 사용자 참여도는 중재 효과를 증진하는 데 중요한 역할을 하지만, 사용자 참여도가 중재 후 건강 결과에 미치는 영향을 살펴본 연구는 부족한 실정이다.

본 연구에서는 예비 조사를 통해 사용 가능성이 입증된 터치케어 시스템을 지역사회 노인을 대상으로 6개월간 적용한 연구결과를 바탕으로 중재 참여도에 따라 대상자를 두 그룹으로 나누어 중재 전후 변화를 비교, 분석하였다. 터치케어 시스템 참여도가 높은 High-engagement (HE)군과 참여도가 낮은 Low-engagement (LE)군으로 나누어 건강 지표로 인지기능, 노쇠, 우울 증상, 영양상태, 낙상 효능감을 측정하여 신체적, 정신적, 인지적 측면에서 종합적으로 중재의 효과를 평가하였다.

### 2. 연구의 목적

본 연구는 IoT 기술을 활용한 노인 대상의 디지털 건강관리 시스템인 터치케어 시스템을 지역사회 노인에게 6개월간 적용한 후, 중재 참여도에 따라 HE군과 LE군으로 나누어 중재 전후 건강 변화를 비교, 분석하는 것을 목적으로 한다. 이를 위해 인지기능, 노쇠, 우울 증상, 영양상태, 낙상 효능감을 주요 건강 지표로 평가하여, 터치케어 시스템이 노인의 신체적, 정신적, 인지적 건강에 미치는 영향을 종합적으로 검토하고자 한다.

### 3. 연구 가설

가설 1: HE군이 LE군에 비해 인지기능, 노쇠, 우울 증상, 영양상태, 낙상 효능감 점수에서 더 향상된 결과를 보일 것이다.

가설 2: HE군이 LE군에 비해 인지기능 평가 도구의 항목별 점수가 향상될 것이다.

## 연구 방법

### 1. 연구 설계

본 연구는 단일군 사전-사후 설계로 진행된 준실험설계 연구 데이터를 활용한 2차 분석 연구이다. 지역사회 노인 50명을 대상으로 터치케어 시스템을 6개월간 적용한 후, 대상자의 참여도에 따라 두 그룹으로 나누어 건강 지표의 변화를 평가하였다. 연구에 등록한 대상자에게 터치 태그 센서, 터치케어 애플리케이션으로 구성된 터치케어 시스템과 상황인지 대화형 인공지능 ‘순이’를 제공하였다. 숙련된 연구원이 대상자의 거주지를 방문하여 터치케어 시스템을 설치하였으며, 연구 시작 전 대상자에게 대면으로 사용 교육을 시행하였다. 터치케어 시스템을 6개월 동안 사용하기 전과 후의 신체적, 정신적, 인지적 건강 상태의 변화를 확인하기 위해 대면으로 설문조사를 실시하였다.

### 2. 연구 대상

본 연구의 대상자는 2022년 용인시에 거주하는 65세 이상 고령자를 모집단으로 하여 용인시 노인복지관, 용인시청, 용인세브란스병원에 대상자 공고문을 부착하여 대상자를 모집하였으며, 총 50명의 대상자가 연구 참여에 동의하였다. 대상자 선정 기준은 본 연구 목적을 이해하고 자발적으로 참여에 서면으로 동의한 65세 이상 노인으로, 스마트폰 사용이 가능하며, 설문조사 응답과 댁내 터치 태그 부착에 동의한 자를 대상으로 하였다. 인지기능 저하나 한국어로 의사소통이 불가하여 스스로 설문에 응대할 능력이 없는 대상자와 거동이 불편하여 연구 참여가 어려운 대상자는 제외하였다.

대상자 수는 G*power 3.0 프로그램을 이용한 반복측정 분산분석에 의해 양측검정, 최소 검정력 .95, 효과크기 .50, 유의수준 .05 일 때 산출된 표본 크기가 42명이나, 중도 탈락률 15%를 고려하여 50명으로 설정하였다[19,20]. 연구 기간에 참여 철회 희망 2명, 골절 1명, 감기로 인한 건강 악화 1명으로 총 4명이 탈락하여 최종적으로 46명이 분석에 포함되었다.

**Figure 1.**
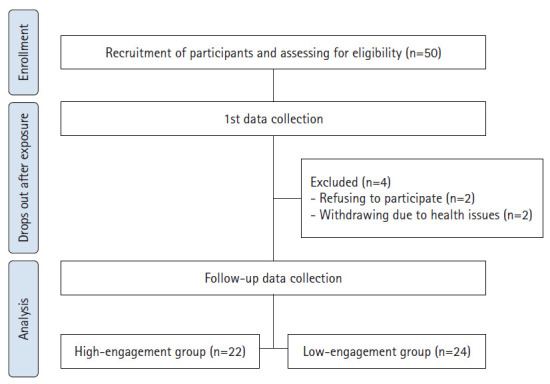
Research flow diagram.

분석에 포함된 46명의 대상자를 중재 시스템 참여도에 따라 두 그룹으로 나누어 중재 전과 후의 건강 상태 변화를 비교하여 평가하였다. 그룹을 나눈 기준은 터치케어 모바일 애플리케이션에 진입한 날짜의 빈도수를 기준으로 하였으며, 중앙값보다 높은 상위 22명을 시스템 참여도가 높은 HE군으로, 중앙값보다 낮은 하위 24명을 시스템 참여도가 낮은 LE군으로 나누어 분석하였다(Figure 1). 본 기준은 디지털 중재 프로그램에 로그인한 횟수가 중재 참여도를 측정하는 정량적 지표로 가장 많이 사용되었다는 선행 연구를 바탕으로 설정하였다[18].

### 3. 연구 도구

본 연구는 대상자의 성별, 나이, 흡연력, 음주력, 운동 여부, 기저질환, 결혼 상태, 교육 정도에 해당하는 일반적 특성을 조사하고, 인지기능 검사와 설문조사를 시행하였다.

#### 1) 인지기능(cognitive function)

경도인지장애를 선별하기 위한 몬트리올 인지 평가를 한국어로 번역한 한국판 몬트리올 인지 평가(Korean version of Montreal Cognitive Assessment, K-MoCA)[21]를 사용하였다. 본 도구는 시공간/집행기능, 이름대기, 기억력, 주의력, 언어기능, 추상력, 지연회상, 지남력에 해당하는 인지 기능을 평가한다. 최고점은 30점으로 점수가 높을수록 인지기능이 양호한 것으로 판단하며, 23점 이상은 정상, 22점 이하는 경도인지장애로 간주한다[21]. 선행 연구에서 번안 도구의 신뢰도는 Cronbach’s α .84이었으며[21], 본 연구에서의 신뢰도는 Cronbach’s α .79이었다.

#### 2) 노쇠(frailty)

대한노인병학회 산하 노인기능평가위원회에서 개발한 한국형 노쇠측정도구(Korean Frailty Index)[22]를 사용하였다. 측정 항목은 8개로 연간 입원 여부, 주관적 건강 상태, 약물복용, 체중감소, 감정 상태, 실금 유무, 보행 능력, 시력 및 청력 불편감으로 구성된다. 점수 범위는 0점에서 8점으로 총점이 높을수록 노쇠 정도가 높음을 의미하며, 0~2점은 정상, 3~4점은 노쇠 전 단계, 5점 이상은 노쇠 단계로 구별한다[22]. 도구 개발 시 신뢰도는 Cronbach’s α .65로 보고되었다[22]. 본 연구에서의 신뢰도는 Cronbach’s α .39이었다.

#### 3) 우울 증상(depressive symptoms)

노인의 우울 증상을 측정하기 위해 15문항으로 구성된 단축형 노인우울척도[23]를 한국어로 번역한 한국판 단축형 노인우울척도(Korean version of the Short Form of Geriatric Depression Scale)[24]를 사용하였다. 총 15문항으로 구성되며, 점수 범위는 0점에서 15점으로 총점이 높을수록 우울 정도가 높음을 의미하고, 절단 점은 8점으로 8점 이상을 우울 정도가 높은 것으로 선별한다[25,26]. 본 연구에서의 신뢰도는 Cronbach’s α .88이었다.

#### 4) 영양상태(nutritional status)

Rubenstein 등[27]이 개발하고, Nestle nutrition institute에서 한국어로 번역하여 제공하는 간이영양사정 측정도구(short-form mini nutritional assessment, MNA-SF)[28]를 사용하였다. 총 6문항으로 식욕 변화, 체중감소 여부, 거동 능력, 스트레스 및 급성 질환 여부, 신경정신과적 질환, 체질량지수를 사정한다. 점수 범위는 0점에서 14점으로 점수가 높을수록 영양상태가 양호하며, 0~7점은 영양불량 상태, 8~11점은 영양불량 위험 상태, 12~14점은 정상상태로 분류한다[28]. 축약형인 본 도구는 원 MNA 도구 점수와 강한 상관관계를 보였으며(r = .95), 영양실조 예측에 대한 민감도 97.9%, 특이도 100.0%, 진단 정확도 98.7%로 영양상태 평가에 적합한 도구로 알려져 있다[27].

#### 5) 낙상 효능감(fall efficacy)

낙상에 대한 두려움을 평가하기 위해 개발된 낙상 효능감 척도[29]를 한국어로 번역하고 타당도와 신뢰도를 검증한 한국어판 낙상 효능 척도(Korean version of Falls Efficacy Scale)[30]를 사용하였다. 총 10문항으로 구성되며, 일상생활에서 필요한 행위를 하는 데 따르는 두려움을 문항 별로 1~10까지 숫자로 응답한다. 낙상에 대한 두려움으로 특정 행위에 전혀 자신감이 없으면 1점, 매우 자신감이 있으면 10점을 기록하며, 점수가 높을수록 낙상에 대한 두려움이 낮음을 의미한다. 선행 연구에서 번안 도구의 신뢰도는 Cronbach’s α .90이었으며[30], 본 연구에서의 신뢰도는 Cronbach’s α .85이었다.

### 4. 터치케어 시스템 중재의 구성

터치케어 시스템은 DNX Co. Ltd에서 개발한 IoT 기반의 통합 건강관리 시스템으로 터치 태그와 터치케어 애플리케이션, 상황인지 대화형 인공지능으로 구성되었다[16]. 본 연구의 대상자는 상황인지 대화형 건강 돌봄 에이전트인 터치케어 시스템을 6개월 동안 사용하였다. 숙련된 연구원이 대상자의 집에 방문하여 자주 이용하는 물건이나 위치에 터치 태그를 직접 부착하고, 대상자의 휴대전화에 터치케어 모바일 애플리케이션을 설치하였다. 설치 후 대면으로 시스템 사용 교육을 시행하였으며, 설치 시점 이후에는 자발적인 사용을 유도하고 관찰하기 위하여 추가 교육이나 모니터링과 같은 연구진의 개입을 최소화하였다.

#### 1) 터치 태그

터치 태그는 대상자가 자주 이용하는 물건(냉장고, 전기밥솥, 리모컨, 좌변기 등)에 부착하여 물리적으로 사물을 만지거나 몸이 닿을 때마다 정보를 수집하였다.

#### 2) 터치케어 모바일 애플리케이션

터치케어 모바일 애플리케이션의 주요 기능은 대상자의 신체 활동 지표 기록, 인지 활동 독려 프로그램, 보호자에게 위험 경보를 전달하는 기능이 있다. 신체 활동 정도는 태그 로그(냉장고, 리모컨, 전자레인지, 약상자 등), 행동 로그(식사, 기상, 복약, 용변, TV 시청 등), 실시간 위치를 기반으로 기록되어 확인할 수 있도록 하였다. 노래 부르기, 영어 퀴즈, 스토리텔링과 같은 간단한 활동을 독려하는 기능과 냉장고 또는 전자레인지 태그 터치 증가, 걸음 수 감소, TV 리모컨 태그 터치 증가를 건강하지 않은 행위로 인식한 후 대상자에게 알리는 기능이 탑재되었다. 위와 같은 행위가 감지되었을 때 강조하여 기록하고, 실시간 행위 그래프를 통해 보호자에게 경보를 전송하였다.

#### 3) 상황인지 대화형 인공지능

상황인식 시스템은 사물 태그의 접촉을 기반으로 고령자의 행동과 상황 데이터를 수집하는 것으로 송신 태그, 수신 앱, 데이터 수집 및 분석용 서버로 구성된다. 돌봄을 제공하는 가상 친구 인공지능 ‘순이’가 고령 대상자의 안전 촉진, 긍정적인 행동 변화 유발, 정서적 외로움 감소를 위해 대상자의 상황적 맥락을 추론하여 메시지를 제공하였다. 모듈은 네 가지로 데이터 인식, 상황인식, 감정 치료, 대화 관리로 구성되었다. 데이터 인식 모듈은 터치 태그에서 활동 정보를 수집하였다. 상황인식 모듈은 정보의 불확실성과 복잡한 환경 요소를 처리하기 위해 확률모델을 사용하여 대상자의 상황에 관한 맥락을 추론하였다. 감정 치료 모듈은 Russell의 활성-변동성 모델(arousal-variance model) [31]을 사용하여 가상 친구인 ‘순이’의 감정 상태를 생성하였다. 대화 모듈은 대상자 맞춤형 메시지를 생성하여 터치케어 애플리케이션에서 음성 메시지를 전달하였다. 이처럼 상황인지 대화형 인공지능은 물건을 만지는 행위나 외출 시간과 같은 다양한 데이터를 기반으로 건강 행동을 촉진하기 위한 음성 메시지 중재를 제공하였다.

### 5. 자료 수집

자료수집은 2022년 8월부터 2023년 3월까지 용인세브란스병원 내 독립적이고 조용한 공간에서 실시되었다. 터치케어 시스템 사용 후 6개월 동안의 건강지표 변화를 확인하기 위하여 중재 전과 후에 시스템 설치에 참여하지 않은 별도의 연구자가 대면 면담으로 인지기능 검사와 설문조사를 수행하였다. 자료 수집은 간호학 석사 과정 대학원생 1명, 간호학 학사를 졸업한 연구원 4명, 임상 연구 간호사 1명이 설문 조사 방법에 대해 충분한 교육을 받은 후 수행하였다.

### 6. 자료 분석

본 연구에서 수집된 자료는 SPSS version 27.0 통계 프로그램(IBM Corp., Armonk, NY, USA)을 사용하여 분석하였으며, 통계적 유의수준은 .05에서 양측 검정 하였다. 자료 분석은 애플리케이션 사용 정도에 따른 그룹 간 차이와 그룹 내 변화를 비교하였다. 종속변수의 정규성은 왜도가 ± 3.0 이하일 때 정규성에 위배되지 않는 것으로 간주하여 모수 검정을 수행하였으며, 정규성 검정을 만족하지 않는 변수는 비모수 검정을 수행하였다. 구체적인 분석 방법은 다음과 같다.

1) 대상자의 일반적 특성 및 시스템 참여도에 따른 그룹 간 동질성은 independent samples t-test, Pearson chi-square test, Fisher’s exact test로 검정하였다.

2) 애플리케이션 참여도에 따른 그룹 내 중재 전과 후 평균의 차이 검정은 paired t-test를 사용하였고, 그룹 간 중재 전과 후 평균의 차이에 대한 비교는 two-way mixed analysis of variance를 사용하여 분석하였다. 낙상 효능감 평가는 그룹 간 independent samples t-test 상 사전 결과에 통계적으로 유의한 차이가 있었으므로, 이를 보정하기 위해 그룹별 사전 결과와 사후 결과의 차이 값을 이용하여 그룹 간 independent samples t-test로 분석하였다.

3) 인지기능 평가의 항목별 세부 분석은 paired t-test로 분석하였으며, HE군과 LE군 모두 이름대기(naming)와 지남력(orientation) 항목의 왜도가 ± 3.0 이상으로 확인되어 정규성 가정을 만족하지 않는 것으로 간주하여 Wilcoxon signed-rank test로 분석하였다.

### 7. 윤리적 고려

본 연구는 용인세브란스병원 윤리심의위원회(IRB)의 승인(IRB No. 9-2021-0124)을 받은 후 수행되었다. 병원 내 독립적인 공간에서 대상자에게 연구 목적, 내용, 절차, 대상자의 권리 및 비밀보장에 대해 구두로 설명하였고, 설명을 이해하였는지 재확인한 이후, 본인의 의사로 결정할 수 있도록 충분한 시간을 제공한 뒤 서면으로 연구 참여 동의서를 받았다.

대상자로부터 측정된 센서 및 위치 정보는 DNX Co. Ltd의 Amazon cloud에 저장되며, 사용이 허가된 자만 비밀번호를 입력한 후 접속이 가능하여 대상자의 개인정보가 노출되지 않도록 하였다. DNX Co. Ltd의 정보관리 담당자는 서버와 cloud 정보가 유출되지 않도록 지속적으로 모니터링하며 보안 상황을 점검하였다. 대상자와 관련된 모든 정보는 식별할 수 없도록 암호화하여 익명성을 보장하였다.

## 연구 결과

### 1. 대상자 특성 및 시스템 참여도에 따른 그룹 간 동질성 검정

대상자의 일반적 특성 및 종속변수에 대한 그룹 간 동질성 검정 결과 일반적 특성에서 두 그룹의 유의한 차이는 나타나지 않았다. 연령은 HE군이 76.27 ± 4.23세, LE군이 76.92 ± 4.55세로 두 그룹 모두 후기 노인이었으며, 여성의 비율은 HE군이 68.2%, LE군이 79.2%로 나타났다. 사전에 측정한 주요 종속 변수에 대한 그룹 간 동질성 검정 결과 인지기능 수준, 노쇠 수준, 우울 증상, 영양상태는 통계적으로 유의한 차이가 나타나지 않았으나, 낙상 효능감 수준은 HE군이 94.45 ± 9.00으로 LE군 84.71 ± 20.72에 비하여 유의하게 높았다(t = −2.10, *p* = .044)(Table 1).

### 2. 시스템 참여도에 따른 그룹 간 중재의 효과 차이 검정

중재에 따른 HE군과 LE군의 주요 종속 변수(인지기능, 노쇠, 우울 증상, 영양 상태, 낙상 효능감)의 그룹 간 차이는 통계적으로 유의하지 않았으므로 가설 1은 기각되었다. 또한, 각 그룹 내에서 중재 전후로 측정된 주요 종속 변수의 평균 점수 변화는 통계적으로 유의한 차이를 보이지 않았다.

중재 전과 후의 그룹별 평균 점수의 변화는 다음과 같다. 인지기능 수준은 HE군은 23.73 ± 4.10에서 24.41 ± 3.61로, LE군은 23.04 ± 3.56에서 22.71 ± 3.98로 나타났다. 노쇠 수준은 HE군은 1.86 ± 1.21에서 1.59 ± 1.44로, LE군은 1.88 ± 1.65에서 1.96 ± 1.57로 나타났다. 우울 증상은 HE군은 2.55 ± 3.74에서 2.09 ± 3.35로, LE군은 3.42 ± 3.60에서 3.33 ± 3.07로 나타났다. 영양상태는 HE군은 12.73 ± 1.52에서 12.73 ± 1.42로, LE군은 12.21 ± 1.47에서 12.46 ± 1.53으로 나타났다(Table 2). 중재 전과 후 낙상 효능감 수준의 그룹 간 차이는 통계적으로 유의하지 않았으며, HE군은 94.45 ± 9.00에서 93.55 ± 9.73으로, LE군은 84.71 ± 20.72에서 89.42 ± 15.85로 나타났다(Table 3).

### 3. 시스템 참여도에 따른 그룹에서 인지기능 검사의 세부 항목별 변화 검정

애플리케이션 참여도가 높은 그룹인 HE군에서 K-MoCA의 7가지 세부 항목 중 시공간/집행기능(visuospatial and executive function)이 사전 평균 점수 3.50 ± 1.14에서 사후 3.95 ± 0.95로 통계적으로 유의하게 향상되어(t = −2.34, *p* = .029), 가설 2는 일부 지지 되었다(Table 4). 항목별 평균 점수는 이름대기(naming)는 2.86 ± 0.47에서 2.82 ± 0.50으로, 주의력(attention)은 5.32 ± 1.32에서 5.09 ± 1.23으로, 언어능력(language)은 2.41 ± 0.80에서 2.23 ± 0.81로, 추상력(abstraction)은 1.41 ± 0.67에서 1.59 ± 0.59로, 지연회상(delayed recall)은 2.27 ± 1.75에서 2.82 ± 1.62로, 지남력(orientation)은 5.95 ± 0.21에서 5.91 ± 0.29로 나타났다. LE군에서는 중재 후 추상력이 1.54 ± 0.78에서 1.08 ± 0.88로 유의하게 감소하였으며(t = 2.89, *p* = .008), 그 외의 항목에서는 유의한 변화가 관찰되지 않았다.

## 논의

본 연구는 준실험설계 연구 데이터를 활용한 2차 분석으로, 대상자의 시스템 참여도를 기준으로 두 그룹으로 나누어 중재 효과의 차이를 평가하였다. 지역사회 노인 46명을 대상으로 IoT 기반의 건강관리 시스템인 터치케어 시스템을 6개월간 적용하여 건강 지표(인지기능, 영양상태, 노쇠, 우울 증상, 낙상 효능감)의 변화를 종합적으로 평가한 결과를 바탕으로, 다음과 같이 논의하고자 한다.

대상자의 애플리케이션 참여도에 따른 그룹 간 중재의 효과를 비교한 결과, HE군과 LE군 간 터치케어 시스템 사용 전과 후에 유의한 차이가 나타나지 않았다. 본 연구에서는 동일한 중재를 받은 대상자들을 시스템 참여도에 따라 두 집단으로 나누어 분석을 수행하였으며, 이러한 연구 설계는 두 집단 간 차이에 대한 낮은 통계적 유의성에 영향을 미쳤을 수 있다. 중재 후 HE군에서 일부 건강 평가 도구(인지기능, 노쇠, 우울 증상)의 평균 점수는 개선되는 경향을 보였으나, 통계적으로 유의하지 않았으므로 임상적 유의성에 관한 추가 연구가 필요하다.

모바일 애플리케이션을 활용한 건강관리 시스템 중재에서 대상자의 참여도를 높이기 위해서는 대상자가 애플리케이션과의 상호작용 기회를 늘릴 수 있도록 중재 빈도를 증가시키는 것이 효과적이다[32]. 특히 대상자의 특성에 맞춘 흥미로운 내용으로 시기 적절하게 제공되는 중재 알림은 대상자에게 참여 동기를 부여하고 수용성을 높여 적극적인 참여를 유도할 수 있다[32]. 본 연구에서는 IoT 기반의 센서를 활용한 상황인지 대화형 인공지능이 대상자의 상황을 인식하여 이에 적합한 알림으로 중재를 제공하였다.

사전 실험으로 진행된 선행 연구에서는 터치케어 시스템의 유용성을 평가한 결과 대상자의 영양상태와 낙상 효능감이 개선되는 효과를 보였다[16]. 본 연구에서 인지기능을 측정하기 위해 사용한 MoCA의 7가지 하위 항목 분석 결과, 애플리케이션 참여도가 높은 HE군에서 시공간/집행기능(visuospatial and executive function)이 유의하게 향상되었다. MoCA는 경도인지장애를 식별하기 위한 도구로서 간이 정신상태검사(Mini-Mental State Examination) 보다 더욱 효과적인 것으로 밝혀졌으며[33], 구체적 인지기능 평가를 위해 하위 항목별로 분석하여 연구에 사용되고 있다[34,35]. 하위 항목 중 시공간/집행기능은 인지기능 상태와 개선을 가장 강력하게 예측하는 지표로 뇌졸중 이후 장기적 인지기능을 예측하는 요인으로 나타났으며[36], 뇌졸중 후 보행 훈련의 효과를 검증한 연구에서 보행 개선 효과를 유효하게 입증하는 신뢰할 수 있는 독립적 도구로 사용되었다[37]. MoCA가 다른 인지기능 측정 도구에 비해 인지 장애를 더 민감하게 선별할 수 있는 차이점이 시공간/집행기능 항목에 있다고 보고되어[38,39], 인지기능을 평가하는 데 있어 시공간/집행기능 영역의 중요성이 높다고 볼 수 있다.

중재 후 HE군에서 시공간/집행기능이 개선된 것은 터치케어 애플리케이션의 기능에 포함된 영어 퀴즈, 글쓰기, 노래하기와 같은 기능의 반복적인 사용과 상황인지 대화형 인공지능의 지속적인 대화 시도가 대상자의 인지기능에 긍정적인 영향을 미쳤을 가능성을 보여준다. 인공지능 로봇을 활용하여 65세 이상 노인을 대상으로 인지기능 관리의 효과를 체계적 문헌 고찰한 선행 연구에서 분석에 포함된 9개 연구의 메타분석 결과 인공지능 로봇이 노인의 인지기능 향상에 효과적인 것으로 확인되었다[40]. 본 연구의 결과는 인공지능을 활용한 중재가 노인의 인지기능 향상에 긍정적인 영향을 미칠 수 있다는 선행 연구를 뒷받침한다.

본 연구는 원거리에서도 노인들이 편리하게 건강관리를 받을 수 있도록 설계된 디지털 건강관리 중재를 통해 건강관리의 접근성을 확대하고자 하였다. 또한, 건강 관리자의 개입을 줄여 노인들이 보편적으로 이용할 수 있는 비용 효율적인 디지털 건강관리 서비스를 구현하였다는 점에서 실용적 가치를 지닌다. 그러나 인지기능 평가 도구의 일부 항목을 제외하고 주요 건강 지표의 유의한 개선이 관찰되지 않았다는 점에서, 인적 개입이 최소화된 접근 방식이 대상자의 건강관리 동기를 충분히 유도하지 못하였을 가능성이 있다. 건강관리 중재 연구에서 노인의 참여를 촉진하기 위해서는 연구팀과 대상자 간의 지속적인 의사소통과 동기 부여가 중요하다는 점에 비추어 볼 때[41], 본 연구의 중재 방식은 동기 부여 측면에서 한계를 가질 수 있다. 국내 5만 3,115명의 노인을 대상으로 허약 개선을 위한 대면, 비대면, 혼합 중재 방식을 비교한 선행 연구에 따르면, 대면 서비스와 인공지능•IoT 기반의 비대면 서비스를 각각 적용한 중재보다 두 방식을 혼합한 중재가 가장 효과적인 것으로 나타났다[42]. 이러한 결과는 건강 제공자의 적극적인 지도, 목표 설정과 동기부여, 성과에 대한 지속적인 피드백이 중재의 효과를 높이는 핵심적 요소임을 보여준다[17]. 따라서 향후 연구에서는 건강관리의 효과를 극대화하기 위해 건강 제공자의 전문적인 지원과 디지털 중재를 병행하는 전략을 통해 대상자의 참여도를 강화하여 중재의 효과성을 높이는 방안이 권장된다.

본 연구는 지역사회 노인을 대상으로 IoT 기반의 건강관리 시스템을 적용한 후, 대상자의 참여도에 따라 두 그룹으로 나누어 중재 효과를 비교하여 분석함으로써 노인의 건강관리 전략 개발에서 참여도의 중요성을 탐구하였다는 점에서 의의가 있다. 다만, 본 연구에는 다음과 같은 제한점이 있다. 첫째, 본 연구는 중재의 적용이 용이하도록 지역 중심으로 대상자를 모집하여 중재의 효과를 확인하였으므로 연구 결과를 확대하여 적용하는데 제한이 있어 추후 다양한 특성을 가진 노인 인구를 포괄하는 후속 연구가 필요하다. 둘째, 본 연구에서는 동일한 중재를 받은 대상자들을 시스템 참여도에 따라 HE군과 LE군으로 나누어 분석을 수행하여 참여도의 중요성을 탐색하였다. 이처럼 동일한 중재를 받은 대상자의 그룹 간 차이를 분석할 시 통계적 유의성의 부재로 이어질 수 있어 중재의 효과를 일반화하는데 한계가 있다. 셋째, 애플리케이션 사용 정도에 따라 대상자를 두 그룹으로 분류할 때, 연구 기간에 모바일 애플리케이션에 진입한 총일수를 분류 기준으로 하였다. 이 기준은 애플리케이션의 활용도를 나타내는 주요 지표로 간주할 수 있으나, 실제 애플리케이션 사용량을 정확하게 반영하지 못할 수 있다. 향후 연구에서는 참여도를 더욱 정밀하게 측정할 수 있는 데이터를 활용할 필요가 있다.

## 결론

본 연구는 지역사회 노인을 대상으로 IoT 기반의 건강관리 모바일 애플리케이션 시스템을 6개월 동안 중재한 후 신체 및 정신 건강과 인지기능 지표를 측정하여 시스템 참여도에 따른 효과를 종합적으로 평가하였다. 터치케어 시스템 사용 후 애플리케이션 참여도가 낮았던 LE군 보다 참여도가 높았던 HE군에서 인지기능 검사 항목 중 시공간/집행기능이 유의하게 향상되었다. 본 연구 결과는 노인 대상의 IoT 기반 디지털 건강관리 시스템이 인지기능 향상에 긍정적인 영향을 미칠 가능성을 보여주며, 이러한 최신 기술을 고령층에서 지속해서 적극적으로 활용할 수 있도록 지원하는 노력이 필요하다. 특히, 사용자의 참여를 촉진하기 위해 건강 관리자의 전문적 지원과 고도의 개인 맞춤형 중재를 적절히 병행하는 것이 효과적인 방안이 될 수 있음을 제안한다.

## Figures and Tables

**Table 1.** Characteristics of the Participants and Baseline Homogeneity Tests (N = 46)

Characteristics		M ± SD or n (%)		Χ^2^ or t	*p*
		HE (n = 22)	LE (n = 24)		
Age (yr)		76.27 ± 4.23	76.92 ± 4.55	0.50	.623^†^
Sex	Men	7 (31.8)	5 (20.8)	0.72	.397^‡^
	Women	15 (68.2)	19 (79.2)		
Marital status	Married	12 (54.5)	8 (33.3)	2.10	.147^‡^
	Others	10 (45.5)	16 (66.7)		
Length of formal education (yr)		12.95 ± 3.62	10.63 ± 5.09	−1.80	.079^†^
Comorbidities	Hypertension	15 (68.2)	15 (62.5)	0.16	.686^‡^
	Diabetes mellitus	7 (31.8)	7 (29.2)	0.04	.845^‡^
	Hyperlipidemia	13 (59.1)	11 (45.8)	0.81	.369^‡^
	Cardiovascular disease	2 (9.5)	4 (16.7)		.670^§^
	Cerebrovascular disease	0 (0)	1 (4.2)		.999^§^
Smoking	Yes	1 (4.5)	1 (4.2)		.999^§^
	No	21 (95.5)	23 (95.8)		
Regular drinking	Yes	7 (31.8)	8 (33.3)		.913^§^
	No	15 (68.2)	16 (66.7)		
Regular exercise	Yes	17 (77.3)	20 (83.3)		.718^§^
	No	5 (22.7)	4 (16.7)		
Dependent variables (baseline)	Cognitive function (K-MoCA)	23.73 ± 4.10	23.04 ± 3.56	−0.61	.547^†^
	Frailty (KFI)	1.86 ± 1.21	1.88 ± 1.65	0.03	.979^†^
	Depressive symptoms (SGDS-K)	2.55 ± 3.74	3.42 ± 3.60	0.81	.425^†^
	Nutritional status (MNA-SF)	12.73 ± 1.52	12.21 ± 1.47	−1.18	.246^†^
	Fall efficacy (K-FES)	94.45 ± 9.00	84.71 ± 20.72	−2.10	.044^†^

M = Mean; SD = Standard deviation; HE = High-engagement; LE = Low-engagement; K-MoCA = Korean version of the Montreal Cognitive Assessment; KFI = Korean Frailty Index; SGDS-K = Korean version of the Short Form of the Geriatric Depression Scale; MNA-SF = Short-Form Mini-Nutritional Assessment; K-FES = Korean version of the Falls Efficacy Scale.

† Independent t-test;

‡ Pearson chi-square test;

§ Fisher exact test.

**Table 2.** Comparison of the Study Variables between HE and LE Groups (N = 46)

Measures	Group	Baseline	After 6 months	Paired t-test	Two-way mixed analysis of variance			
		M ± SD		t (*p*)	Group	Time	G×T	Effect size
					F (*p*)	F (*p*)	F (*p*)	(η^2^_p_)
Cognitive function (K-MoCA)	HE (n = 22)	23.73 ± 4.10	24.41 ± 3.61	−1.48 (.155)	1.26 (.267)	0.21 (.646)	1.82 (.184)	.04
	LE (n = 24)	23.04 ± 3.56	22.71 ± 3.98	0.57 (.573)				
Frailty (KFI)	HE	1.86 ± 1.21	1.59 ± 1.44	0.88 (.389)	0.23 (.636)	0.27 (.609)	0.94 (.338)	.02
	LE	1.88 ± 1.65	1.96 ± 1.57	−0.40 (.692)				
Depressive symptoms (SGDS-K)	HE	2.55 ± 3.74	2.09 ± 3.35	1.27 (.219)	1.19 (.282)	0.79 (.379)	0.38 (.543)	.01
	LE	3.42 ± 3.60	3.33 ± 3.07	0.18 (.863)				
Nutritional status (MNA-SF)	HE	12.73 ± 1.52	12.73 ± 1.42	< 0.01 (> .999)	1.11 (.298)	0.30 (.589)	0.30 (.589)	.01
	LE	12.21 ± 1.47	12.46 ± 1.53	−0.92 (.366)				

HE = High-engagement; LE = Low-engagement; M = Mean; SD = Standard deviation; G×T = Group×Time interaction; K-MoCA = Korean version of the Montreal Cognitive Assessment; KFI = Korean Frailty Index; SGDS-K = Korean version of the Short Form of the Geriatric Depression Scale; MNA-SF = Short-Form Mini-Nutritional Assessment.

**Table 3.** Comparison of Fall Efficacy between the HE and LE Groups (N = 46)

Measure	Group	Baseline	After 6 months	Baseline−After 6 months	t (*p*)
Fall efficacy (K-FES)	HE (n = 22)	94.45 ± 9.00	93.55 ± 9.73	0.91 ± 8.30	−1.74 (.091)
	LE (n = 24)	84.71 ± 20.72	89.42 ± 15.85	−4.71 ± 13.28	

Values are presented as the mean ± standard deviation. HE = High-engagement; LE = Low-engagement; K-FES = Korean version of Falls Efficacy Scale.

**Table 4.** Changes in the Subscale Scores of the K-MoCA among the HE and LE Groups (N = 46)

		HE group (n = 22)				LE group (n = 24)			
K-MoCA domain	Possible range	Baseline	6 months	t or Z	*p*	Baseline	6 months	t or Z	*p*
Visuospatial and executive function	0-5	3.50 ± 1.14	3.95 ± 0.95	−2.34	.029^†^	3.79 ± 1.14	3.46 ± 1.32	1.40	.175^†^
Naming	0-3	2.86 ± 0.47	2.82 ± 0.50	−0.58	.564^‡^	2.75 ± 0.68	2.63 ± 0.58	3.00	.180^‡^
Attention	0-6	5.32 ± 1.32	5.09 ± 1.23	1.10	.285^†^	4.75 ± 1.29	5.08 ± 1.10	−1.78	.088^†^
Language	0-3	2.41 ± 0.80	2.23 ± 0.81	1.45	.162^†^	2.08 ± 0.78	2.38 ± 0.82	−1.50	.148^†^
Abstraction	0-2	1.41 ± 0.67	1.59 ± 0.59	−1.07	.296^†^	1.54 ± 0.78	1.08 ± 0.88	2.89	.008^†^
Delayed recall	0-5	2.27 ± 1.75	2.82 ± 1.62	−1.92	.069^†^	2.17 ± 1.52	2.17 ± 1.61	< 0.01	> .999^†^
Orientation	0-6	5.95 ± 0.21	5.91 ± 0.29	−0.58	.564^‡^	5.96 ± 0.20	5.92 ± 0.28	2.00	.564^‡^

Values are presented as the mean ± standard deviation. HE = High-engagement; LE = Low-engagement; K-MoCA = Korean version of the Montreal Cognitive Assessment.

† Paired t-test;

‡ Wilcoxon signed-rank test.

## Data Availability

Please contact the corresponding author for data availability.
